# Antioxidant Capacities and Analysis of Phenolic Compounds in Three Endemic *Nolana* Species by HPLC-PDA-ESI-MS

**DOI:** 10.3390/molecules200611490

**Published:** 2015-06-22

**Authors:** Mario J. Simirgiotis, Julio Benites, Carlos Areche, Beatriz Sepúlveda

**Affiliations:** 1 Laboratorio de Productos Naturales, Departamento de Química, Facultad de Ciencias Básicas, Universidad de Antofagasta, Av. Coloso S-N, Antofagasta 1240000, Chile; 2 Facultad de Ciencias de la Salud, Universidad Arturo Prat, Iquique 1100000, Chile; E-Mail: juliob@unap.cl; 3 Departamento de Química, Facultad de Ciencias, Universidad de Chile, Casilla 653, Santiago 7800024, Chile; E-Mail: areche@uchile.cl; 4 Departamento de Ciencias Químicas, Universidad Andres Bello, Campus Viña del Mar, Quillota 980, Viña del Mar 2520000, Chile; E-Mail: bsepulveda@uc.cl

**Keywords:** Chilean plants, Atacama Desert, phenolics, antioxidant capacity, HPLC-MS, Paposo Valley, *Nolana*

## Abstract

The antioxidant features, polyphenolic composition and chromatographic fingerprints of the aerial parts from three Chilean endemic plants from the Paposo Valley located on the cost of the Atacama Desert were investigated for the first time using high pressure liquid chromatography coupled with photodiode array detector and electrospray ionization mass analysis (HPLC-PDA-ESI-MS) and spectroscopic methods. The phenolic fingerprints obtained for the plants were compared and correlated with the antioxidant capacities measured by the bleaching of the DPPH radical, the ferric reducing antioxidant power (FRAP) and quantification of the total content of phenolics and flavonoids measured by spectroscopic methods. Thirty phenolics were identified for the first time for these species, mostly phenolic acids, flavanones, flavonols and some of their glycoside derivatives, together with three saturated fatty acids (stearic, palmitic and arachidic acids). *Nolana ramosissima* showed the highest antioxidant activity (26.35 ± 1.02 μg/mL, 116.07 ± 3.42 μM Trolox equivalents/g dry weight and 81.23% ± 3.77% of inhibition in the DPPH, FRAP and scavenging activity (SA) assays, respectively), followed by *N. aplocaryoides* (85.19 ± 1.64 μg/mL, 65.87 ± 2.33 μM TE/g DW and 53.27% ± 3.07%) and *N. leptophylla* (124.71 ± 3.01, 44.23 ± 5.18 μM TE/g DW and 38.63% ± 1.85%).

## 1. Introduction

**Figure 1:**
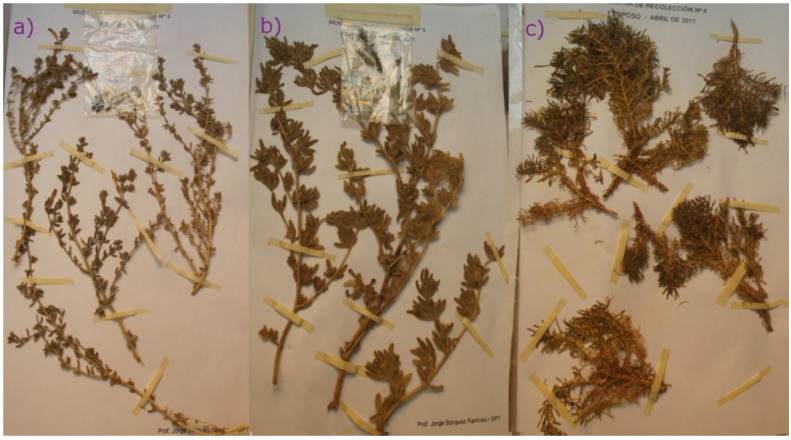
Pictures of herbarium specimens of (**a**) *Nolana leptophylla*, (**b**) *N. aplocaryoides* and (**c**) *N. ramosissima* collected in Paposo Valley, II region of Chile.

Polyphenols are one of the most important groups of compounds widely present in fruits and vegetables. High consumption of vegetables and fruits has been associated with a lowered incidence of degenerative, chronic and incurable diseases [1,2,3]. The protective effects of polyphenols against the induction of cellular and tissue impairment, mainly generated under oxidative stress conditions, can be attributed to direct scavenging activities against reactive species of oxygen (ROS), as well as to the induction of cell responses that activate the antioxidant effect at intracellular levels involved in cellular metabolism and cellular survival [1,4]. The analysis of polyphenol content in a complex organic matrix is a difficult task, and HPLC with photodiode array detectors is one of the most useful techniques [5,6,7,8]. Mass spectrometry with HPLC-PDA has undergone significant technological improvements in the last few years, especially with the development of interfaces, such as ElectroSpray (ESI) or Atmospheric Pressure Chemical Ionization (APCI) and several mass detectors, such as Time of Flight (TOF) OrbiTrap (OT) or Ion Trap (IT). Among all those techniques, several antioxidant phenolics in edible plants [9], fruits [5,7,10,11,12], nuts [13] and food byproducts [14] were analyzed using HPLC with Photodiode Array Detectors coupled to Ion Trap Mass analyzers thorough an ESI interface (HPLC-PDA-ESI-IT-MS). The genus *Nolana* comprises about 89 endemic species restricted to fog-dependent Lomas formations of coastal Peru and Chile [15]. So far, little is known about the chemistry of *Nolana* species. *Nolana sedifolia* was investigated for phenolic compounds and its activity against the fungus *Botrytis cinerea* [16]. However, mostly labdane diterpenoids were reported, to the best of our knowledge, from *Nolana*. From *Nolana elegans*, three new labdane diterpenoids were reported more than ten years ago [17], as well as two labdanes were reported from *Nolana rostrata* [18] and two more from *N. filifolia* [19]. Furthermore, from *N. coelestis*, four sesquiterpenoids were reported [20]. The aim of the present work is the chemical analysis of some important native Chilean *Nolana* plants (Figure 1) from a protected area of the Atacama Desert, in northern Chile (Paposo Valley), and the comparison of the antioxidant properties and total phenolics measured by spectroscopy. The polyphenolic fingerprints and polyphenolic content were compared and correlated with the antioxidant capacities measured by the bleaching of the DPPH radical, the ferric reducing antioxidant power (FRAP) and the superoxide anion scavenging activity assay (SA). The compounds in the plants were identified for the first time with the help of PDA analysis and mass spectrometry (HPLC-PDA-ESI-MS) plus a comparison with authentic standards.

## 2. Results and Discussion

### 2.1. MS-PDA Identification of Polyphenolics in Three Nolana Species from Northern Chile

**Figure 2:**
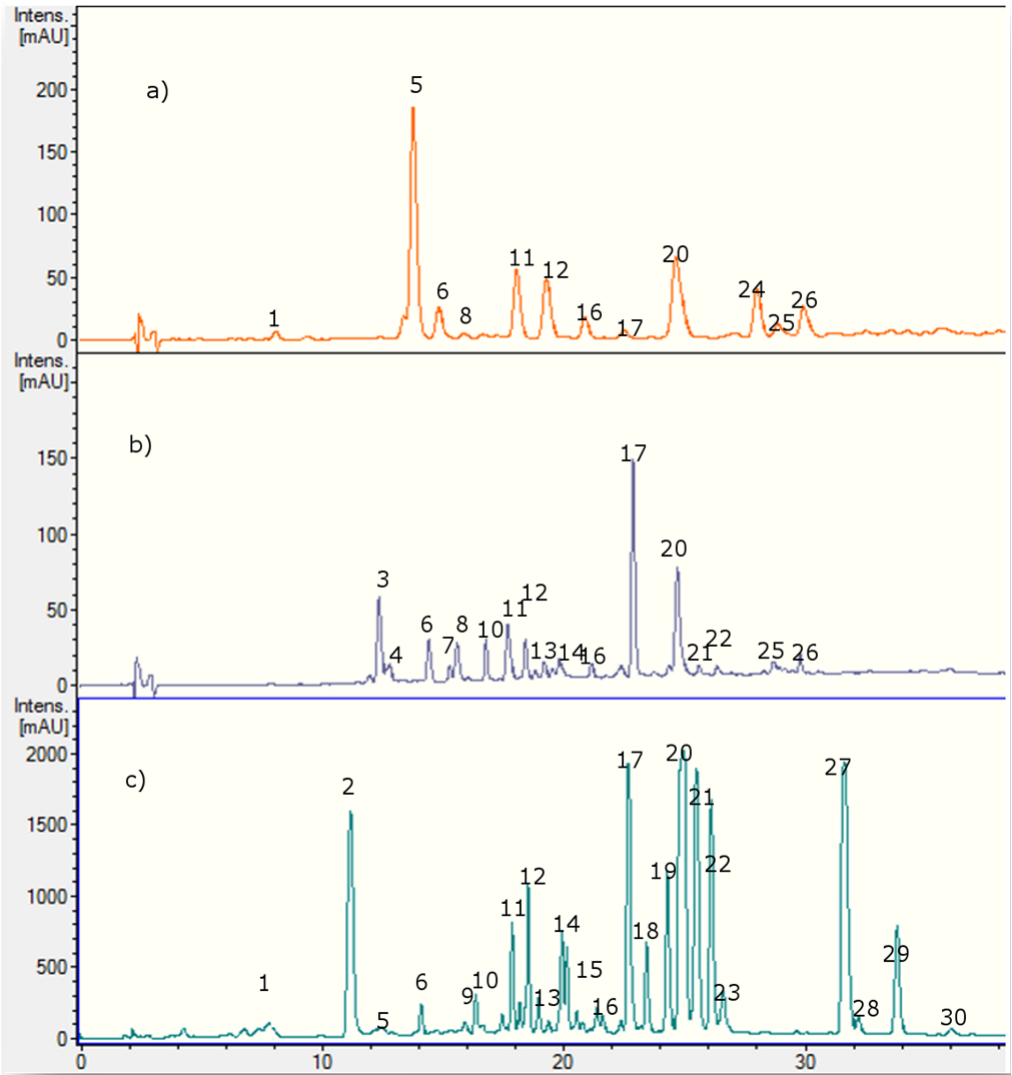
HPLC-UV chromatograms at 280 nm of (**a**) *Nolana leptophylla*, (**b**) *N. aplocaryoides* and (**c**) *N. ramosissima* collected in Paposo Valley, II region of Chile.

**Figure 3:**
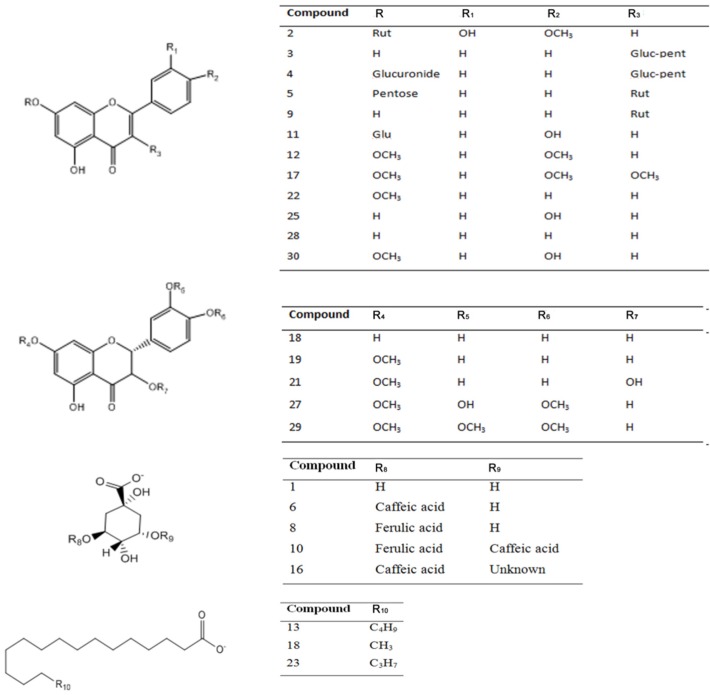
Structures of the main compounds detected in *Nolana leptophylla*, *N. aplocaryoides* and *N. ramosissima* collected in Paposo Valley, II region of Chile.

**Figure 4:**
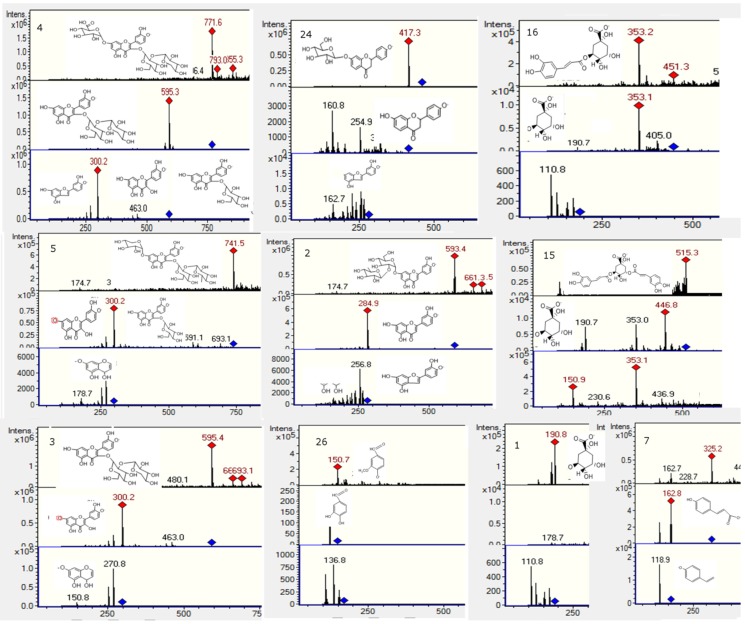
Structures, full scan MS and MS^n^ spectra of Peaks **1**, **2**, **3**, **4**, **5**, **7**, **15**, **16**, **24** and **26**.

**Figure 5:**
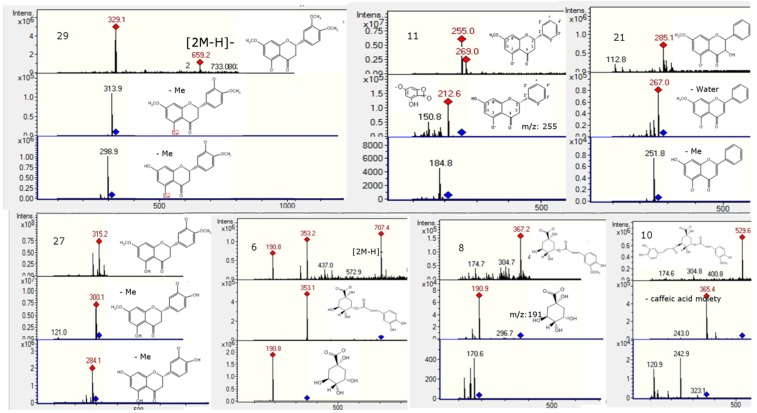
Structures, full scan MS and MS^n^ spectra of Peaks **6**, **8**, **10**, **11**, **21**, **27** and **29**.

**Figure 6:**
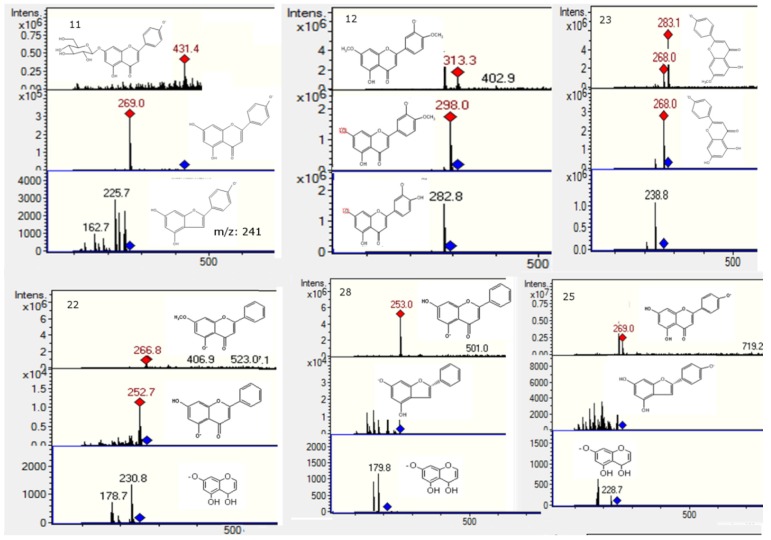
Structures, full scan MS and MS^n^ spectra of Peaks **11**, **12**, **22**, **23**, **25** and **28**.

**Figure 7:**
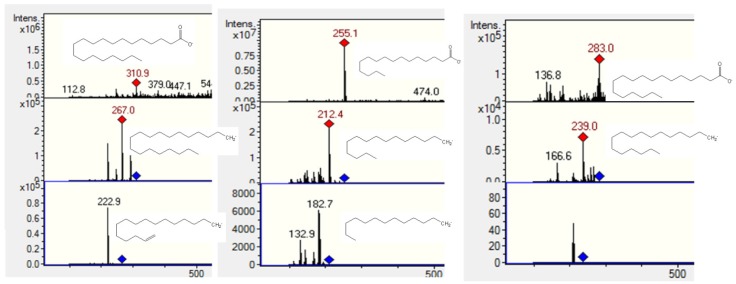
Structures, full scan MS and MS^n^ spectra of Peaks **13**, **18** and **23**.

Several polyphenolics in *Nolana* species were detected and identified using HPLC with UV-visible data (PDA, Figure 2, Table 1) and ion trap electrospray mass spectrometry (IT-ESI-MS, Table 2). The 30 compounds identified in *Nolana ramosissima*, *N. aplocaryoides* and *N. leptophylla* were mainly flavones, flavanones, phenolic acids, fatty acids, some glycoside flavonoid conjugates and their derivatives. Twenty one compounds were detected in *Nolana ramosissima*, (Peaks **1**, **2**, **5**, **6**, **9**–**23** and **27**–**29**), nine in *N. leptophylla* (Peaks **1**, **6**, **11**, **16**, **17**, **20**, **24**, **25** and **26**) and fourteen in *N. aplocaryoides*, (Peaks **3**, **4**, **6**, **7**, **8**, **10**–**17**, **20** and **21**) (Figure 2, Table 2), respectively. Below is the detailed explanation and identification of the compounds using HPLC and PDA and MS^n^ analysis plus co-elution with some of the authentic available standards. Figure 2 shows the HPLC fingerprints at 280 nm, Figure 3 the structures of the main compounds detected and Figure 4, Figure 5, Figure 6 and Figure 7 full MS and MS^n^ spectra of several compounds detected.

**Table 1:** HPLC-PDA-ESI-MS^n^ data of *Nolana leptophylla*; *N. aplocaryoides* and *N. ramosissima* ethyl acetate extracts.

Peak Number	Retention Time (min)	UV Max	M-Ion (ppm)	Other Ions (Aglycon Moiety)	Identification	Plant
1	7.9	198	191	178, 173, 148, 110	Quinic acid	Lepto, ramo
2	11.3	350, 260	593	285 [M − H − rutinose]^−^, 253, 179, 151	Luteolin-7-*O*-rutinose	ramo
3	12.1	350, 254	595	463 (quercetin 3-*O*-glucoside), 301(quercetin-1H), 300 (quercetin-2H), 179, 151	Quercetin-3-*O*-glucosyl-pentoside	aplo
4	12.3	350, 254	771	595 (quercetin-3-*O*-hexosyl-hexoside), 463 (quercetin 3-*O*-glucoside), 301 (quercetin), 179, 151	Quercetin-3-*O*-glucosyl-pentoside-7-*O*-glucuronide	aplo
5	12.8	350, 254	741	609 ([M − H − xylose]^−^, 301 [M − H − rutinose − xylose]^−^,179, 151	Quercetin-7-*O*-xyloside-3-*O*-rutinoside	ramo
6	14.0	310, 246	353	707 [2M − H]^−^, 191 (quinic acid)	Chlorogenic acid *	Lepto, ramo, aplo
7	14.7	324, 275	325	651 [2M − H]^−^, 163 [M − H − glucose]^−^, 119[M − H − glucose − CO_2_]^−^	p-Coumaric acid glucoside	aplo
8	15.3	310, 247	367	191 (quinic acid)	Feruloyl-quinic acid	aplo
9	16.0	350, 254	609	1219 [2M − H]^−^, 301 [M − H − rutinose]^−^, 179, 151	Rutin *	ramo
10	16.3	310, 246,	529	367 ([M − H − caffeic acid moiety]^−^	Feruloyl-caffeoyl-quinic acid	Aplo, ramo
11	18.0		431	269 (apigenin)	7-*O*-glucosyl-apigenin	Lepto, ramo, aplo
12	18.7	350, 260	313.3	298, 282	4′,7′ -dimethoxyluteolin	Lepto, ramo, aplo
13	19.0	207	311	267 [M − H − CO_2_]^−^, 223 [M − H − CO_2_ − H_2_O]^−^	Arachidic acid *	ramo, aplo
14	20.0	275	441	305, 175, 147 (cinnamic acid moiety)	Cinnamic acid derivative	aplo
15	20.2	310, 240	515	353, 141	Dicaffeoyl-quinic acid	ramo
16	21.8	310, 246	451	353 [chlorogenic acid − H]^−^, 191 [quinic acid]^−^	Chlorogenic acid derivative	Lepto, ramo, aplo
17	22.9	334, 275	327	312 [M − H − CH_3_]^−^,297 314 [M − H − 2CH_3_]^−^	5-hydroxy-3,4′7 trimethoxy-flavone *	Lepto, ramo, aplo
18	23.5	207	255	212 [M − H − CO_2_]^−^, 182 [M − H − CO_2_ − H_2_O]^−^	Palmitic acid *	ramo
19	24.6	292, 330 sh	255	213, 183, 172	Pinocembrin *	Ramo
20	25.2	291, 330 sh	269, 271	255[M − CH_3_]^−^, 213	Pinostrobin *	Lepto, ramo, aplo
21	25.8	292, 330 sh	285, 287	267[M − H_2_O]^−^, 251 [M − H_2_O − CH_3_]^−^	3,5-dihydroxy-7-methoxy-flavanone	ramo, aplo
22	26.0	334, 270	267	253 [M − H − CH_3_]^−^, 231, 179, 151	chrysin-7-methyl ether	ramo
23	26.9	207	283	239 [M − H − CO_2_]^−^	Stearic acid *	ramo
24	28.1	275, 215 29.1 sh	417	255 [M − H − glucose]^−^	Liquiritin	lepto
25	29.1	334, 269	269	240, 182, 179, 151	Apigenin *	lepto
26	29.6	310, 28 sh	151	136 [M − CH_3_]^−^	Vanillin *	lepto
27	31.9	285	315	300 [M − 2H − CH_3_]^−^, 284 [M − 2H − 2CH_3_]^−^	Hesperetin 7-*O*-methyl ester	ramo
28	32.1	334, 270	253	179, 151	Chrysin	ramo
29	33.9	292, 330 sh	329	659 [2M − H]^−^, 314 [M − H − CH_3_]^−^, 299 [M − H − 2CH_3_]^−^	5-hydroxy-3′4′7 trimethoxy-flavanone *	ramo
30	36.0	334, 269	283, 285	268, 238	Apigenin-7-O-methyl ether	ramo

Abbreviations: lepto, *Nolana leptophylla*; aplo, *N. aplocaryoides*; ramo, *N. ramosissima* EtOAc; * identified by spiking experiments with authentic compounds.

### 2.2. Flavonoids and Derivatives

MS profiling of Peak **2** produced a parent ion at *m*/*z* 593 (Figure 4) and daughter ions at *m*/*z* 285 [M − H − rutinose]^−^, 257, 179 and 151, corresponding to luteolin aglycone and its fragment ions, revealing the compound to be luteolin-7-*O*-rutinoside [21]. Peak **3** (Figure 4) was identified as quercetin-3-*O*-glucosyl-pentoside (595, and MS^n^ ions at *m*/*z* 463, 301, 179 and 151) [22]. Peak **4** with a [M − H]^−^ ion at *m/z* 771, producing daughter ions at 595 (loss of 176 mass units, a glucuronide moiety), 463 (quercetin-3-*O*-glucoside) and 301 (quercetin, with MS^3^ 179 151), was identified tentatively as quercetin-3-*O*-glucosyl-pentoside-7-*O*-glucuronide (Figure 4). Likewise, Peak **5** with a [M − H]^−^ at *m/z* 741 and MS^n^ ions at *m/z* 609 (rutin, by loss of xylose or the rutinose moiety, 132 u), 591 (rutin−H_2_O), 301 (quercetin), 179 and 151 was tentatively identified as a rutin pentoside (Figure 4) [23]. Peak **9** was identified as rutin ([M − H]^−^ ion at *m/z* 609, MS at 301, 179 and 151) by co-elution with an authentic standard. Peak **10** (Figure 5) with a [M − H]^−^ parent ion at *m/z* 529, which yielded a feruloylquinic acid ion at *m/z* 365, was identified as a feruloyl-caffeoylquinic acid derivative. Peak **11** (Figure 5) presented a pseudomolecular ion at *m/z* 431, which experienced a hexoside loss (162 u) to produce an apigenin ion at *m/z* 269 and, thus, was identified as 8-*O*-glucosyl-apigenin. This compound was identified with a spiking experiment with the authentic compound. Peak **12** showed UV maxima at 268 and 346 nm, characteristic for 3′,4′ dimethoxyflavones [10]. This peak showed precursor ions at *m/z* 315 [M + H]^+^ and at *m/z* 313 [M − H]^−^. The MS/MS spectrum in negative ionization mode showed product ions at *m/z* 298 and at *m/z* 283, formed after elimination of methyl groups and probably indicating a dimethoxyluteolin. These data led to identification of this peak as 4′,7′ -dimethoxyluteolin (4′,5-dihydroxy-3′,7-dimethoxyflavone) [11,24], which was also compared with an authentic compound. Peak **20** with a [M − H]^−^ ion at *m/z* 269 and a daughter fragment at *m/z* 255 ([M − H − CH_3_]^−^) was identified using co-elution with an authentic standard of pinostrobin, which was isolated from this plant previously by us, and its structure was elucidated by single-crystal X-ray analysis [25]. In the same manner, Peak **19** was identified as the related compound pinocembrin (5,7-dihydroxy-flavanone). A related compound with different UV-VIS data (λ max 292, 330), Peak **21** (Figure 5), showed a [M − H]^−^ ion at *m/z* 285 and MS^1^ ion at *m/z* 267 ([M − H − H_2_O]^−^) and 251 ([M − H − H_2_O − CH_3_]^−^). This compound was identified as 3,5-dihydroxy-7-methoxyflavanone by spiking experiments with the authentic compound. Peak **22** (Figure 6) with a pseudomolecular ion at *m*/*z* 267 and daughter fragment at *m*/*z* 253 ([M − H − CH_3_]^−^, chrysin) was identified as 5′hydroxy-7-methoxyflavone or chrysin-7-methyl ether. Peak **23** (Figure 6) with a pseudomolecular ion at *m/z* 283 and an MS^1^ ion at *m/z* 268 was identified as 4,5-dihydroxy-7-methoxyflavone or apigenin 7-methyl-ether standard. Peak **24** with [M − H]^−^ at *m/z* 417 and an MS^1^ ion at *m/z* 255 (liquiritigenin) was identified as the glucosylated flavanone liquiritin [26]. Peak **25** (Figure 6) showed [M − H]^−^ ions at *m/z* 269 and produced daughter ions at *m*/*z* 240, 182 and 151 (substituted Rings A and B) and was identified as apigenin. Peak **27** (Figure 5) showed a [M − H]^−^ ion at *m*/*z* 315 and MS^n^ ions at *m*/*z* 300 and 284 produced by the loss of two methyl groups, which pointed to the presence of a dimethoxylated flavanone and, thus, was tentatively identified as 3′,5-dihydroxy-4′,7-dimethoxyflavanone, the identity being confirmed with the compound previously isolated from the plant. Peak **28** showed a [M − H]^−^ ion at *m*/*z* 253 and fragments characteristic of Rings A and B of dihydroxyflavones and was identified as chrysin. Likewise, Peak **29** showed a [2M − H]^−^ adduct ion at *m/z* 659 and [M − H]^−^ ion at *m/z* 329, which produced daughter ions at *m/z* 314 and *m/z* 299, which evidenced the loss of two methyl groups from the parent 329 ion and, consequently, was tentatively identified as 5-hydroxy-3′,4′,7-trimethoxy-flavanone, the identity being confirmed with the compound previously isolated from the plant. In the same manner, a related flavone (UV max 254, 354 nm) identified with Peak **17** showed a pseudomolecular ion at *m/z* 327, which produced daughter ions at *m/z* 312 and *m/z* 297, which evidenced the loss of two methyl groups from the parent ion and was identified as 5-hydroxy-3,4′,7-trimethoxyflavone. Peak **30** showed a pseudomolecular ion at *m/z* 283 producing a fragment at 268 (M-methyl group) and was tentatively identified as apigenin methyl ester [27].

### 2.3. Fatty Acids

Peaks **13**, **18** and **23** (Figure 7) were identified as saturated fatty acids. They showed [M − H]^−^ ions at *m*/*z* 283, 255 and 311 [28] and neutral CO_2_ losses at *m*/*z* 238, 212 and 267, respectively, and were thus characterized as stearic, palmitic and arachidic acids. Further identification of the compounds was performed by TLC comparison with authentic samples (Silica gel F_254_ TLC plates, Merck, Darmstadt, Germany, with the solvent system n-hexane-ethyl acetate 7:3 v/v and developed with a solution of vanillin 1% in ethanol and 10% sulfuric acid and heating).

### 2.4. Phenolic Acids and Related Compounds

Peak **1** with a [M − H]^−^ ion at *m/z* 191 was identified as quinic acid (Figure 5). Peak **6** (Figure 5) was identified as one of the known chlorogenic acids, caffeoylquinic acid (molecular anion at *m*/*z* 353, MS^2^ at *m*/*z* 191 and 173 (191−H_2_O) by comparison with an authentic sample [29]). Peak **7** showed a [2M − H]^−^ adduct ion at *m/z* 651 and [M − H]^−^ ion at *m/z* 325, which produced daughter ions at *m/z* 163 (loss of glucose) and *m/z* 119 (decarboxylated coumaric acid) and, thus, was tentatively identified as coumaric acid glucoside [30,31]. Peak **8** (Figure 5) with a [M − H]^−^ ion at *m/z* 367 yielding a quinic acid ion at *m/z* 191 by loss of a ferulic acid moiety was identified as feruloyl-quinic acid. Peak **15** (Figure 4) with a pseudomolecular ion at *m/z* 515 and MS^n^ ions at *m/z* 353 (caffeoyl-quinic acid) and *m/z* 191 (quinic acid) was identified as dicaffeoylquinic acid [32]. Peak **26** with a pseudomolecular ion at *m/z* 151 was tentatively identified as vanillin. This compound was previously reported to occur in the related plant *Nolana sedifolia* [16].

### 2.5. Unknown Compounds

Peak **14** with a UV max corresponding to cinnamic acid (Table 1) with a parent ion at *m/z* 44,1 which yielded product ions at *m/z* 305, 175 and 147 (cinnamic acid), was identified as an unknown cinnamic acid derivative. Peak **16** with a [M − H]^−^ ion at *m/z* 451 yielding an MS^1^ ion at *m/z* 353 (caffeoyl-quinic acid or chlorogenic acid) and MS^2^ ions at *m/z* 191 (quinic acid) and *m/z* 179 (caffeic acid) was identified as an unknown chlorogenic acid derivative.

### 2.6. Total Phenolics and Flavonoids Contents

**Figure 8:**
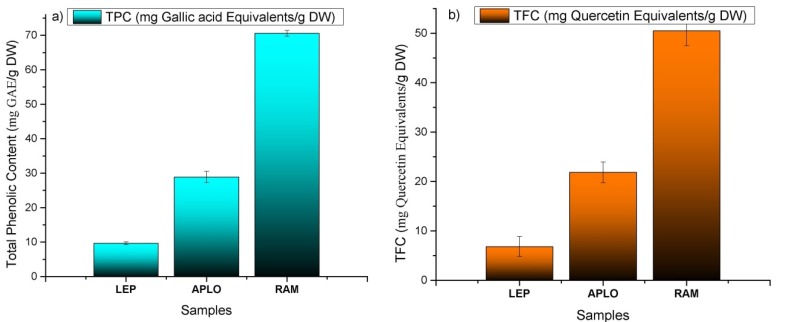
(**a**) Total phenol content (TPC) and (**b**) total flavonoid content (TFC) of *Nolana leptophylla* (LEP), *N. aplocaryoides* (APLO) and *N. ramosissima* (RAM) collected in Paposo Valley, II region of Chile.

The total phenolic content (TPC; Figure 8a) varied from 9.66 ± 0.34 for *N. leptophylla* to 70.50 ± 0.80 mg Gallic acid (GA) equivalents/g DW for *N. ramosissima* and showed linear correlation with the antioxidant assays (R^2^ = 0.990 and R^2^ = 0.999 for the TPC/DPPH and TPC/FRAP assays, respectively); the TPC of our sample of *N. ramosissima* showed values 1.5-times higher than a blueberry extract, but was close to that reported for an extract of Chilean Calafate *Berberis microphylla* [29] and was three times the value reported for an extract of strawberry leaves [8]. The total flavonoid content (TFC; Figure 8b) showed a similar trend, varying from 6.82 ± 0.20 for *N. leptophylla* to 50.50 ± 0.66 mg quercetin/g DW for *N. ramosissima*. The TFC showed a linear correlation with the antioxidant assays (R^2^ = 0.995 for TFC/DPPH and R^2^ = 0.997 for the TFC/FRAP assays, respectively). The total flavonoid content for our sample of *N. ramosissima* was five-times more than the Chilean *Adesmia emarginata* [33], but half of that presented by the aerial parts of *Buddleja globosa* [33].

### 2.7. Antioxidant Features

**Figure 9:**
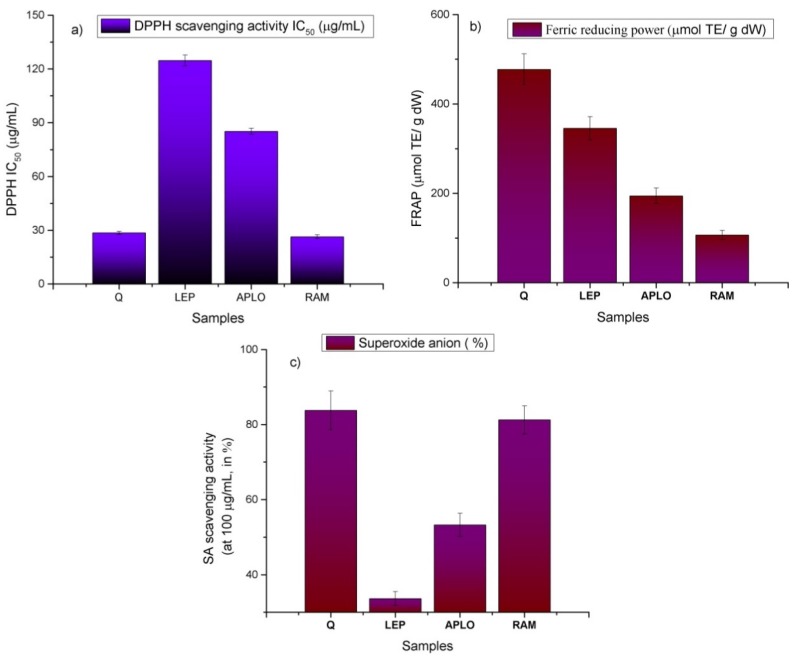
(**a**) DPPH scavenging activity, (**b**) ferric reducing antioxidant power and (**c**) Superoxide anion scavenging activity of *N. leptophylla* (LEP), *N. aplocaryoides* (APLO) and *N. ramosissima* (RAM) from the II region of Chile.

The antioxidant capacity cannot be evaluated using a single test according to several authors [34]. Thus, three commonly-used assays, differing in their working principles (Figure 9), were employed in this work, using the DPPH method (Figure 9a), the ferric-reducing antioxidant power (FRAP; Figure 9b) and the superoxide anion scavenging activity assay (SA; Figure 9c). The DPPH assay is based on the hydrogen donating capacity to scavenge DPPH radicals, while the FRAP assay is an electron transfer-based test measuring the substance ability to reduce Fe^3+^ to Fe^2+^ [35]. The superoxide anion scavenging properties of a compound reduces the speed of the appearance of a formazan chromophore formed in a specific reaction using the xanthine-xanthine oxidase system [36]. In this work, the order of the antioxidant activity measured by the bleaching of the radical DPPH and the ferric reducing antioxidant power (FRAP) shown by the three *Nolana* plants was *Nolana ramosissima* > *N. aplocaryoides* > *N. leptophylla*. A similar trend was observed for superoxide anion scavenging activity. Indeed, *N. ramosissima* showed the highest antioxidant activity (26.35 ± 1.02 μg/mL, 116.07 ± 3.42 μM Trolox equivalents (TE)/g dry weight and 81.23% ± 3.77% of inhibition in the DPPH, FRAP and SA assays, respectively (Figure 9), followed by *N. aplocaryoides* (85.19 ± 1.64 μg/mL, 65.87 ± 2.33 μM TE/g DW and 53.27% ± 3.0%), and *N. leptophylla* (124.71 ± 3.01, 44.23 ± 5.18 μM TE/g DW and 38.63% ± 1.85%, Figure 9). The bleaching of the radical DPPH for *N. ramosissima* extract was close to that shown by the standard quercetin (28.53 ± 0.89 μg/mL, respectively). The antioxidant activities showed a positive correlation with polyphenolic content assays (0.990 ≥ R^2^ ≥ 0.999). Superoxide anion scavenging activity showed the best activity for *N. ramosissima* (81.23% ± 3.77% tested at 100 μg/mL), which was close to that presented by quercetin (83.77% ± 5.15% at 100 μg/mL). SA activity showed also good correlations with the DPPH (R^2^ 0.990) and FRAP assays (R^2^ 0.998).

## 3. Experimental Section

### 3.1. Chemicals and Plant Material

Folin-Ciocalteu phenol reagent (2 N), reagent grade Na_2_CO_3_, AlCl_3_,HCl, FeCl_3,_NaNO_2,_ NaOH, quercetin, trichloroacetic acid, sodium acetate, HPLC-grade water, HPLC-grade acetonitrile, reagent grade MeOH and formic acid were obtained from Merck (Darmstadt, Germany); several fatty acids, quercetin, pinocembrin (all standards with purity higher than 95% by HPLC) were purchased either from ChromaDex (Santa Ana, CA, USA), Extrasynthèse (Genay, France) or Wuxi apptec Co. Ltd. (Shangai, China). Gallic acid, TPTZ (2, 4, 6-Tris(2-pyridyl)-s-triazine), Trolox, tert-butyl-hydroperoxide, nitro blue tetrazolium, xanthine oxidase and DPPH (1,1-diphenyl-2-picrylhydrazyl radical) were purchased from Sigma-Aldrich Chemical Co. (St. Louis, MO, USA). *Nolana leptophylla* (Miers) I. M. Johnst. sp. leptophylla, *Nolana aplocaryoides* (Gaudich.) I.M. Johnst and *Nolana ramosissima* I.M. Johnst were collected at Paposo Valley II region, northern Chile in April 2011. Voucher herbarium specimens were deposited at the Laboratorio de Productos Naturales, Universidad de Antofagasta, Antofagasta, Chile, with the numbers Nl-111004-1, Na-111004-1, Nr-111004-1, respectively. Sampling was performed using sterile disposable gloves and rigid plastic sample containers, and each sample was submitted individually by overnight courier to our laboratory in Antofagasta to prevent deterioration, dried under dark and milled at ambient temperature in our laboratory in Antofagasta. This sampling methodology was previously used for other Chilean samples [5,7,37].

### 3.2. Sample Preparation

Ten grams of each dried plant were finally pulverized in a mortar, defatted thrice with 100 mL of n-hexane and then extracted with 100 mL of ethyl acetate in the dark in an ultrasonic bath for one hour each. The extracts were combined, filtered and evaporated *in vacuo* in the dark (40 °C). The solutions were combined and evaporated to dryness under reduced pressure (40 °C) to give 416.51, 467.30 and 499.93 mg of *Nolana leptophylla*, *N. aplocaryoides* and *N. ramosissima* extracts, respectively.

### 3.3. Liquid Chromatography Analysis

A portion of each extract (approximately 2.5 mg) obtained as explained above was dissolved in 2 mL 0.1% HCl in MeOH, filtered through a 0.45-μm micropore membrane (PTFE, Waters) before use and was injected (20 μL) into the HPLC-PDA and ESI-MS equipment.

### 3.4. Mass Spectrometric Conditions

An Esquire 4000 Ion Trap mass spectrometer (Bruker Daltoniks, Bremen, Germany) was connected to an Agilent 1100 HPLC instrument via an ESI interface for HPLC-ESI-MS analysis. Full scan mass spectra were measured between *m/z* 150 and 2000 μ in positive ion mode and negative ion mode for all compounds. High purity nitrogen was used as the nebulizer gas at 27.5 psi, 350 °C and at a flow rate of 8 L/min. The mass spectrometric conditions for negative ion mode were: electrospray needle, 4000 V; end plate offset, −500 V; skimmer 1, −56.0 V; skimmer 2, −6.0 V; capillary exit offset, −84.6 V; and the operating conditions for positive ion mode were: electrospray needle, 4000 V; end plate offset, −500 V; skimmer 1, 56.0 V; skimmer 2, 6.0 V; capillary exit offset, 84.6 V; capillary exit, 140.6 V. Collisionally-induced dissociation (CID) spectra were obtained with a fragmentation amplitude of 1.00 V (MS/MS) using ultrahigh pure helium as the collision gas.

### 3.5. Antioxidant Assays

#### 3.5.1. Free Radical Scavenging Capacity

The free radical scavenging capacity of the extracts was determined by the DPPH ^.^assay as previously described with some modifications [8,34]. Briefly, 50 μL of extract or pure compound prepared at different concentrations were added to 2 mL of a fresh 0.1 mM solution of DPPH in methanol and allowed to react at 37 °C in the dark. After thirty minutes, the absorbance was measured at 517 nm. The DPPH scavenging ability as a percentage was then calculated as: DPPH scavenging ability = (A_control_ − A_sample_/A_control_) × 100. Afterwards, a curve of % DPPH bleaching activity *versus* concentration was plotted, and IC_50_ values were calculated. IC_50_ denotes the concentration of sample required to scavenge 50% of DPPH free radicals. The lower the IC_50_ value, the more powerful the antioxidant activity. Quercetin (from 15.0 to 250.0 μg/mL, R^2^ = 0.999) was used as the standard antioxidant compound.

#### 3.5.2. Ferric Reducing Antioxidant Power

The determination of ferric reducing antioxidant power or ferric reducing ability (FRAP assay) of the extracts was performed as previously described [29,38]. Quantification was performed using a standard calibration curve of antioxidant Trolox (from 0.2 to 2.5 μmol/mL, R^2^: 0.995). Samples were analyzed in triplicate, and results are expressed in μmol TE/gram dry mass.

#### 3.5.3. Superoxide Anion Scavenging Activity

The enzyme xanthine oxidase is able to generate superoxide anion radical (O_2_^**•**−^) ‘*in vivo*’ by oxidation of reduced products from intracellular ATP metabolism. In the reaction, the superoxide anion generated by the enzyme reduces nitro blue tetrazolium (NBT) to a blue formazan. The absorbance of the formazan produced was determined at 560 nm. Superoxide anion scavengers reduce the speed of generation of the chromophore. The superoxide anion scavenging activities of isolated compounds and fractions were measured spectrophotometrically in a microplate reader as reported previously, and the absorbance at 560 nm was recorded for 60 s (formation of blue formazan) [36,37,39]. The standard flavonoid and the EtOAc extracts were evaluated at 100 μg/mL. Values are presented as the mean ± the standard deviation of three determinations. The percentage of superoxide anion scavenging effect was calculated as follows:

% of scavenging activity: (E − S)/E × 100, where E = A − B and S = C − (B + D)

where A is the optical density of the control; B is the optical density of the control blank; C is the optical density of the sample; and D is the optical density of the sample blank.

#### 3.5.4. Polyphenol and Flavonoids Contents

The total polyphenolic contents (TPC) of *Nolana* extracts were determined by the Folin-Ciocalteu method [6,7,40] with some modifications. An aliquot of each crude EtOAc extract (200 μL, approximately 2 mg/mL) was added to the Folin-Ciocalteu reagent (2 mL, 1:10 v/v in purified water), and after 5 min of reaction at room temperature (25 °C), 2 mL of a 100 g/L solution of Na_2_CO_3_ were added. Sixty min later, the absorbance was measured at 710 nm. The calibration curve was performed with gallic acid (concentrations ranging from 16–500 μg/mL, R^2^ = 0.999), and the results were expressed as mg gallic acid equivalents/g dry mass. Determination of total flavonoid content (TFC) of the extracts was performed as reported previously [41] using the AlCl_3_ colorimetric method. Quantification was expressed by reporting the absorbance in the calibration graph of quercetin, which was used as a standard (from 0.1–65.0 μg/mL, R^2^ = 0.994). Results are expressed as mg quercetin equivalents/g dry weight. All spectrometric measurements were performed using a Unico 2800 UV-VIS spectrophotometer (Shangai, Unico instruments, Co, Ltd, Shangai, China) or a Pharospectroquant 300 (Merck, Darmstadt, Germany).

### 3.6. Statistical Analysis

The statistical analysis was carried out using the originPro 9.0 software packages (Originlab Corporation, Northampton, MA, USA). The determination was repeated at least three times for each sample solution. Analysis of variance was performed using ANOVA. Significant differences between means were determined by a Tukey comparison test (*p*-values <0.05 were regarded as significant).

## 4. Conclusions

Thirty compounds, including three fatty acids (Peaks **13**, **18** and **23**), six phenolic acids or derivatives (Peaks **6**–**8**, **10**, **14** and **16**), six flavones (Peaks **12**, **17**, **22**, **24**, **25**, **28** and **30**) and five flavanones (Peaks **19**–**21**, **27** and **29**) were identified in three *Nolana* species from the Chilean Desert in the II region of Chile using ESI-MS for the first time. Among the 30 compounds identified in the three plants under study, twenty three compounds were detected in *N. aplocaryoides*, fourteen in *N. leptophylla* and nine in *N. ramosissima*. Indeed, *N. ramosissima* showed the most complex polyphenol profile, while the other *Nolana* species showed a simpler pattern. However, *N. ramosissima* presented the highest antioxidant features and polyphenolic content followed by *N. aplocaryoides*, which makes this plant the better candidate for industrial crop production and potential use in functional foods and nutraceuticals.
